# Evaluation of the Rosa Chatbot Providing Genetic Information to Patients at Risk of Hereditary Breast and Ovarian Cancer: Qualitative Interview Study

**DOI:** 10.2196/46571

**Published:** 2023-09-01

**Authors:** Elen Siglen, Hildegunn Høberg Vetti, Mirjam Augestad, Vidar M Steen, Åshild Lunde, Cathrine Bjorvatn

**Affiliations:** 1 Western Norway Familial Cancer Center Department of Medical Genetics Haukeland University Hospital Bergen Norway; 2 Faculty of Health Studies VID Specialized University Bergen Norway; 3 Department of Clinical Science University of Bergen Bergen Norway; 4 Department of Global Public Health and Primary Care University of Bergen Bergen Norway

**Keywords:** chatbot, chatbots, genetic, trust, acceptability, perception, perceived, genetic counseling, hybrid health care, digital health tool, digital information tool, digital health technology, virtual assistant, hereditary breast and ovarian cancer, hereditary, genetic testing, technology, genetic clinic, digital tool, ovarian cancer, breast cancer, information retrieval, women’s health, breast, ovarian, cancer, oncology, mobile phone

## Abstract

**Background:**

Genetic testing has become an integrated part of health care for patients with breast or ovarian cancer, and the increasing demand for genetic testing is accompanied by an increasing need for easy access to reliable genetic information for patients. Therefore, we developed a chatbot app (Rosa) that is able to perform humanlike digital conversations about genetic BRCA testing.

**Objective:**

Before implementing this new information service in daily clinical practice, we wanted to explore 2 aspects of chatbot use: the perceived utility and trust in chatbot technology among healthy patients at risk of hereditary cancer and how interaction with a chatbot regarding sensitive information about hereditary cancer influences patients.

**Methods:**

Overall, 175 healthy individuals at risk of hereditary breast and ovarian cancer were invited to test the chatbot, Rosa, before and after genetic counseling. To secure a varied sample, participants were recruited from all cancer genetic clinics in Norway, and the selection was based on age, gender, and risk of having a BRCA pathogenic variant. Among the 34.9% (61/175) of participants who consented for individual interview, a selected subgroup (16/61, 26%) shared their experience through in-depth interviews via video. The semistructured interviews covered the following topics: usability, perceived usefulness, trust in the information received via the chatbot, how Rosa influenced the user, and thoughts about future use of digital tools in health care. The transcripts were analyzed using the stepwise-deductive inductive approach.

**Results:**

The overall finding was that the chatbot was very welcomed by the participants. They appreciated the 24/7 availability wherever they were and the possibility to use it to prepare for genetic counseling and to repeat and ask questions about what had been said afterward. As Rosa was created by health care professionals, they also valued the information they received as being medically correct. Rosa was referred to as being better than Google because it provided specific and reliable answers to their questions. The findings were summed up in 3 concepts: “Anytime, anywhere”; “In addition, not instead”; and “Trustworthy and true.” All participants (16/16) denied increased worry after reading about genetic testing and hereditary breast and ovarian cancer in Rosa.

**Conclusions:**

Our results indicate that a genetic information chatbot has the potential to contribute to easy access to uniform information for patients at risk of hereditary breast and ovarian cancer, regardless of geographical location. The 24/7 availability of quality-assured information, tailored to the specific situation, had a reassuring effect on our participants. It was consistent across concepts that Rosa was a tool for preparation and repetition; however, none of the participants (0/16) supported that Rosa could replace genetic counseling if hereditary cancer was confirmed. This indicates that a chatbot can be a well-suited digital companion to genetic counseling.

## Introduction

### Background

Chatbots, which are artificial intelligence (AI)–powered computer programs that can engage in conversation with users, have for long existed as information support tools both in private companies and public settings [1]. Lately, chatbots are increasingly used in health care services [2]. Indeed, 2 of the current postulated top trends in health care are hybrid care models combining internet-based and in-person services and the digitalization of health care specialties [3].

The Rosa chatbot was created to perform humanlike digital conversations about hereditary breast and ovarian cancer, and its development and testing process has been described previously [4]. Rosa is built on a commercially available platform supporting Norwegian language, using machine learning and natural language processing. It has a database of predefined answers about hereditary breast and ovarian cancer written by genetic counselors and geneticists from all health regions in Norway. This means that Rosa does not construct any answers itself, ensuring trustworthy information, which is particularly important in health care. If a question is asked without a matching answer, the chatbot will provide a fallback answer saying it does not understand the question. A mobile app and a web application programming interface were created to facilitate interaction between the patient and the natural language processor. The mobile app (Rosa) serves as the interface for the patients, and the level of AI is limited to understanding the patients’ questions and selecting the right answers. Rosa can be downloaded to the patient’s smartphone and contains functions such as chat, read more buttons, learning videos, links to relevant websites, and general information about BRCA-related cancers [4]. The intention is to support patients and their relatives throughout the process of genetic counseling and, possibly, genetic testing for hereditary breast and ovarian cancer. However, personalized communication is not included; for instance, Rosa does not provide genetic test results or remind users about follow-up appointments. It merely provides quality-assured information tailored to this patient group’s specific needs based on extensive user and usability testing [4].

Hereditary breast and ovarian cancer are among the most prevalent forms of hereditary cancer syndromes, accounting for approximately 2% to 5% of breast cancers and 15% to 20% of ovarian cancers [5]. Having hereditary cancer has implications beyond the individual affected, extending to their relatives who must make decisions about their own paths in terms of genetic testing, surveillance, and possible preventive measures. There is a need for digital tools to support the various steps of genetic testing and counseling [6]. In medical genetics, chatbots are already used to assist different aspects, such as identifying patients at risk of hereditary colorectal cancer syndromes [7], collecting family health history [8], and collecting and providing genomic information before or after pretest consultations [9]. So far, the application of chatbots in genetic services has been well accepted by patients [10], and it has the potential to improve family communication and help genetic counselors and other clinicians to scale their services [11]. By providing clear and concise explanations of the results and their implications, they can help patients to understand their genetic test results [9]. In particular, chatbots might be helpful for patients with poor health literacy [12] and patients who are geographically isolated, with limited access to traditional genetic services. Furthermore, they can be beneficial for patients who wish to engage in information privately or anonymously [13].

Previous studies point to the empowering potential in digital health tools [14,15]. Given the diversity and potential embedded in chatbot technology as an advanced digital tool, chatbots may have the potential to empower patients [16] and, in turn, may positively influence treatment compliance [15]. Empowerment is widely understood as a process of helping people to assert control over the factors that affect their health [17]. Within psychiatry, AI bots seem to have great potential in managing psychiatric symptoms, augmenting therapeutic treatments [18], and providing support [19]. During the COVID-19 pandemic, users of the SimSimi chatbot, a large open-domain social chatbot, sought health-related information and shared emotional messages. This indicates that chatbots may provide accurate health information and emotional support [20]. This is consistent with the development of Rosa [4], thus indicating the possibility of Rosa positively influencing patients’ empowerment.

### Objectives

The responsibility to ensure that AI-generated technology in health care is used in an equitable and appropriate manner, upholding their fundamental values—better health and wellness for all—lies with researchers, experts, stakeholders, and users of the technology [21]. Thus, the increasing emergence of chatbots in health care calls for a thorough documentation of the trust and usefulness of the technology [22].

Therefore, the objectives of this study were to (1) explore the perceived utility and trust in chatbot technology among healthy patients at risk of hereditary breast and ovarian cancer and (2) explore how interaction with a chatbot regarding information about hereditary breast and ovarian cancer influences the patients.

## Methods

### Participants

This project is a collaboration between all familial cancer centers in Norway, located in Bergen, Oslo, Trondheim, and Tromsø. After signing the agreement protocols, the centers started inviting healthy patients at risk of breast and ovarian cancer to use Rosa during the genetic counseling and predictive testing process. We invited 175 patients from all health regions in Norway, who were referred for genetic counseling either based on knowledge about a BRCA1/2 mutation in the family or based on relevant cancer history in the family that is sufficient to trigger genetic testing of BRCA1/2, to download the Rosa app and use it as they wished. In general, the participants were invited to this study via letter 1 to 2 weeks before the pretest counseling session, but some were invited during the pretest counseling owing to missing precounseling invitation. No material other than the Rosa app was provided as a part of the project. All participants had access to genetic counseling. They were further asked to consent to individual in-depth interviews via video.

Among the 175 invited individuals, 61 patients consented to individual in-depth interviews by returning the consent form. Participants were consecutively selected based on age, gender, geographical location, and genetic risk setting to ensure that our final interview sample was reflecting the total group. To schedule an individual video interview, 29 of the patients were contacted via telephone. Of these 29 patients, 4 declined to be interviewed, 6 did not answer the phone, 1 was excluded because of poor Norwegian language skills, and 1 did not attend the interview appointment. In addition, 1 interviews had to be cancelled, as the participant had not actually used the chatbot. Finally, 16 interviews were conducted with participants who were carefully assembled.

### Data Collection

We chose a qualitative approach using in-depth interviews that allowed us to explore how people experience interacting with a chatbot and gave us the opportunity to further improve the chatbot based on their feedback. A semistructured interview guide focusing on usability, perceived usefulness, trust, how Rosa influenced the user, and thoughts about future use of digital tools in health care was followed, also allowing each respondent to freely speak about topics of their own choice regarding using the chatbot (refer to Multimedia Appendix 1 for the English translation of the semistructured interview guide). The interviews were conducted by 2 authors (ES and MA), transcribed by the first author, and listened to and proofread by 2 coauthors (MA and ÅL). The transcripts were copied into NVivo (QSR International) for coding and analysis preparation. Owing to the COVID-19 pandemic, all interviews were conducted via video. To mimic a private conversation as much as possible, video was chosen over telephone interviews. A video platform approved for patient consultations was used for this purpose, but consistent with the approval given by the ethics committee, only the soundtrack was recorded.

### Data Analysis

Data analyses were performed by the research group (all authors except VMS), consisting of 1 clinical geneticist (HHV), 2 genetic counselors (ES and CB), 1 health scientist (ÅL), and 1 nurse specialist (MA). ÅL and MA were invited into the project owing to their special competence in qualitative research.

**Figure 1:**
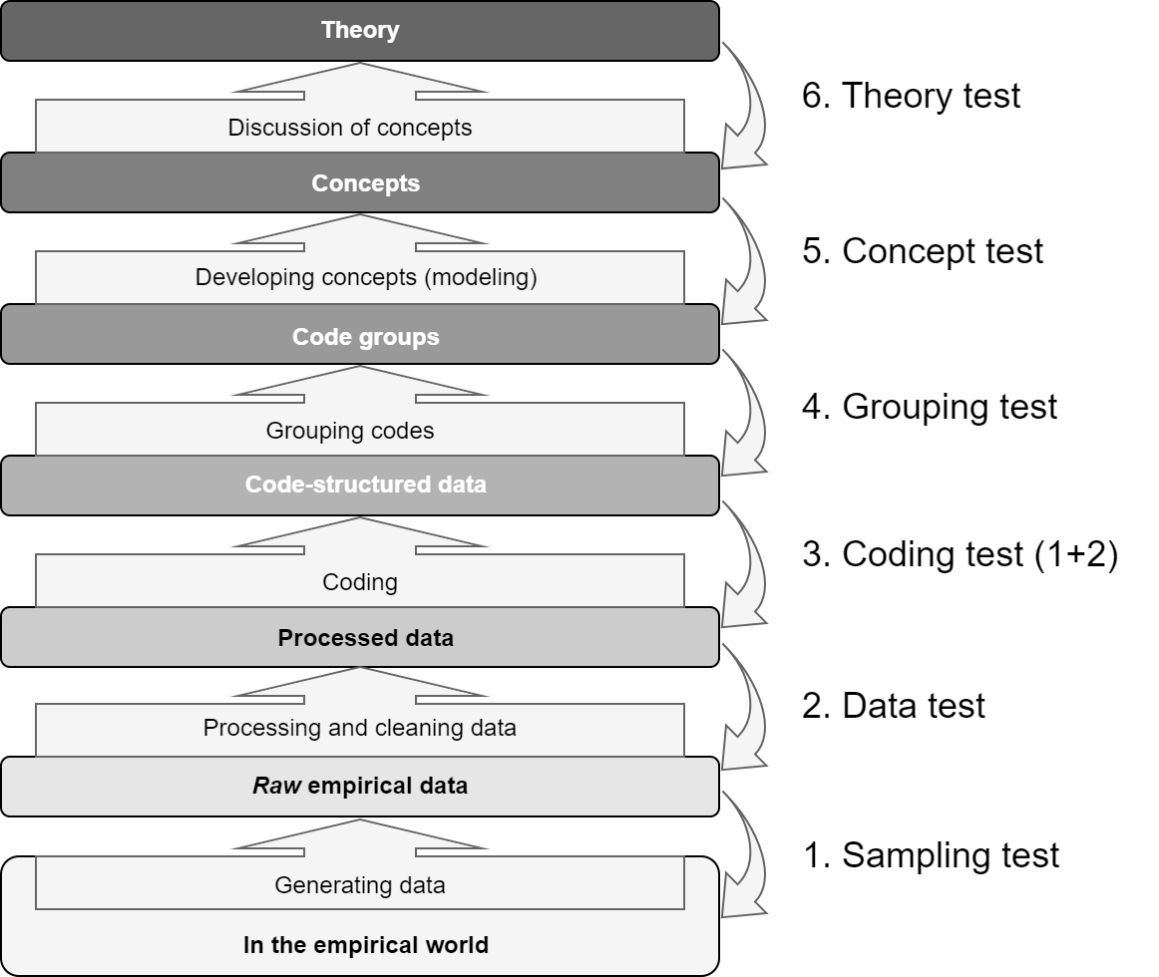
Illustrating the stepwise-deductive inductive (SDI) model. This is described as a 5-step process, because step 6 is not always applicable or possible. (Reproduced from Tjora [23], with permission from Aksel Tjora).

We chose the stepwise-deductive inductive approach in the data analysis, as described by Tjora [23]. This model presents a research process in which detailed data analysis leading to the development of concepts is central. It is based on an inductive principle, which begins with raw data and moves toward concepts or theories through incremental deductive feedback loops. Therefore, it is called the “stepwise-deductive inductive” (SDI) approach (Figure 1). This approach directs researchers to focus on the analysis phase of their study and lets the empirical data define the codes. Through the SDI process, researchers work their way inductively from data to theory and deductively go back in every step to match the theoretical data with the empirical data, aiming at developing concepts or theories.

### Analytical Process

The 2 initial steps (ie, sampling test and data test) in the 5-step SDI analytical process resulted in selective recruitment of 1 man and 2 women who carry a BRCA1/2 mutation among the final 16 participants. All transcripts were read by 3 coauthors (CB, MA, and ÅL). Step 3 of the SDI process, which is the first round of coding, resulted in 75 codes, deductively validated by the same coauthors, ensuring that all codes were empirically based and could not have been made in advance. Overall, 2 codes were discarded in this step: “I have used the app as I was asked to” and “Previous experiences with chatbots.” The first code confirms app use by all participants and is the premise for the interview. The second code is not an empirical code; thus, it is not a valid code according to the SDI approach. Of the remaining codes, 15 codes were registered only once and 6 codes were registered in only 1 transcript but several times in the same transcript, leaving us with 21 codes unique to 1 person. All other codes (52 codes) were repeated in 2 to 11 transcripts, between 2 to 21 times each.

Step 4 started with rereading the transcripts aiming at adding codes that were missing. No codes were added. The codes were then grouped thematically. Initially, this left us with 13 thematic groups (several of which were clearly overlapping) and 1 group of single codes in this step. Further reading and discussion in the research group narrowed it down to 6 groups. We had 3 research group meetings to complete this step, and codes were moved back and forth among groups following the deductive process of ensuring that all codes were grouped into thematically exclusive groups—different from the other groups, yet internally consistent. All the codes could be placed in either group.

Step 5 started with rereading the transcripts, codes, and all coded sentences. In this step, the number of transcripts and number of times each code appeared were given weight in each group, ensuring that the most frequent codes are reflected in the naming of the final concepts.

### Ethics Approval

The Regional Committee for Medical and Health Research Ethics in Western Norway (project number 2019/763) approved this study.

### Informed Consent and Privacy

All participants signed an informed consent form. They were also given the opportunity to call a genetic counselor on duty every day, if they had any questions regarding the study, written information, or content of the chatbot. A major challenge and concern when using machine intelligence per se is the privacy consideration. A chatbot has the potential to learn a lot about an individual, such as their habits, worries, medical challenges, and psychological well-being. It is highly important that this information does not reach unauthorized individuals. In building Rosa, we chose a platform that safeguards these issues [4] and performed an extensive risk and vulnerability analyses.

## Results

### Chatbot Use During the Study Period

The individual participant’s specific chatbot use was not recorded owing to privacy regulations; recording of only overall use was approved. There were 195 entries to the app during the study period. Of these 195 entries, 35 (17.9%) were mere downloads where the app was opened but not explored. Log-in time where the app was explored ranged from 30 seconds to 20 minutes. We registered 884 commands in the app during the study period, with an average of 5 commands per entry. Of these 884 commands, as many as 724 (81.9%) were predefined clicks, such as suggestions for “read more,” leaving us with 160 (18.1%) commands that were open-ended questions. Rosa was unable to provide answers to 53.1% (85/160) of the open-ended questions. Owing to the high level of predefined clicks in the app, where 724 commands were predefined questions leading to predefined correct answers, the overall fallback rate was 9.7%.

### Participant Characteristics

Among the 16 participants interviewed, 2 were men and 14 were women, with age ranging from 20 to 55 years. Of the 16 participants, 8 revealed during the interview that they knew about a person who carries a BRCA1/2 mutation in the near family, hence having 25% to 50% risk of carrying the family mutation. Among these 8 participants, 3 had been informed that they had inherited the mutation, 2 did not know yet, and 3 had received a negative test result. The remaining participants (8/16) had no known mutation in the family and, eventually, received a negative genetic test result. The interviews lasted from 20 to 40 minutes. Only 1 of the 16 participants stated in the interview to have had downloaded the app before seeing the genetic counselor.

### Emerging Concepts

#### Overview

During the interviews almost all the participants (13/16) commented that they felt Rosa would have been a helpful tool to have had before the first counseling session, as a means to prepare them for what lies ahead. Their evaluation of Rosa concerns their thoughts about its value throughout the genetic process and as a tool outside the time frame in which they have used it themselves.

Step 5 of the analytical process led to the development of 3 concepts describing the patients’ experiences with using Rosa: “Anytime, anywhere”; “In addition, not instead”; and “Trustworthy and true.” An elaboration of what these concepts entail is given in the following sections.

#### Anytime, Anywhere

Rosa’s accessibility emerged as one of our key findings very early in the analytical phase. The 24/7 availability was highlighted by several participants (13/16). A participant explained that if one thinks about something in the middle of the night, they could ask Rosa immediately instead of waiting for phone hours at the hospital. This indicates that it is not only anytime but also anywhere that makes Rosa attractive:

I enjoyed having information this way. You can communicate with someone when you want to, not just when you have the appointment with the counselor. You can use this all the time.

Participant 7

The setting that all the information regarding the genetic test was gathered in this app made it easy for them to get answers to their questions. As one does not always know what questions to ask, Rosa contains read more suggestions after every answer provided. All participants (16/16) mentioned this function as very smart and helpful. The user can then keep reading about a subject guided by the chatbot, instead of thinking about relevant questions to ask in a topic they had little knowledge about in advance.

A participant said that it was almost similar to a public awareness tool with its easy access and quick answers. The participant stated that Rosa was an information tool in which one can actively engage, regardless of whether it is early in the morning or late at night. All participants (16/16) mentioned the immediacy and availability as a very important and attractive asset. This gave them time to reflect about the information and process and, consequently, helped them to ask more precise questions specific to their own situation.

We asked one of the participants whether she had had any expectations in terms of what this app could contribute. She said the following:

I didn’t have access to this app before the first counseling session, so I had to search for information myself online to find stuff. But when I had this app I found it much easier, because everything was in one place.

Participant 8

Some of the participants referred to Google as an alternative way of getting information when one has unanswered questions. In that comparison, Rosa was mentioned as a tool to get answers quickly, yet specific to one’s question, providing the user with the information they need and not the information they do not need:

What’s good about this? You don’t have to Google.

Participant 6

As Google provides one with potentially thousands of hits, many of which will be in English, several of our participants (13/16) experienced challenges with using Google for this purpose, and Rosa was highlighted as a provider of that *1* answer the user needs.

#### In Addition, Not Instead

A wish to have access to both the chatbot and to a genetic counseling session was expressed by all participants (16/16). Most of them (12/16) agreed that this app was best suited as a supplement to and not as a replacement of genetic counseling. Furthermore, they stressed that Rosa could not replace a conversation but held that it was more suited as an alternative to internet searches.

There is a lot of information during genetic counseling. Then it was really good to use this app and read afterwards. It repeated what you had heard. And you can read it several times. I liked that.

Participant 14

Rosa became a tool that helped them in the genetic counseling process, supplementing the situation. They enjoyed having this app to read and repeat what they had heard during the counseling session as many times as they felt necessary. A participant who claimed that Rosa could have replaced the pretest counseling was asked how she would feel if she was left with only Rosa and no counseling at all. She thought for some time and said that if hereditary cancer had been confirmed, this app would not have sufficed. Others also confirmed this when asked if Rosa could replace genetic counseling. Many of them (9/16) stressed that a conversation, via phone or video or face to face, would be preferable in addition to Rosa, to have a place to ask questions. The combination of genetic counseling and having access to Rosa was especially mentioned as a comforting combination, making the situation less scary, not more:

The combination of counseling and chatting with Rosa made the situation less scary.

Participant 5

A participant felt that having access to Rosa comforted her and made her more confident in the process she was about to initiate. The fact that an app such as this exists was a reminder that others are in the same situation, otherwise, it would not have been made.

Some of the participants said that they would have liked to have access to Rosa before the genetic counseling session, so that they could have prepared themselves better. A participant who had downloaded Rosa few days before the pretest counseling session confirmed that the information provided by Rosa did not make her anxious or worried; on the contrary, she claimed to be calm before meeting the genetic counselor because she had some basic information and knew, to some extent, what she was facing. The information that she received through Rosa made the situation less difficult for her. Other participants had similar opinions and said that they would have liked to have read about hereditary cancer by using Rosa before coming to counseling. They argued that this would have made it possible to prepare some questions before counseling. In this way, they would be able to ask more specific or tailored questions suited for their own situation to a larger extent:

If I had had this app before the counseling session I would have known more and could have asked more personal questions to her [the counselor].

Participant 8

Several participants (8/16) mentioned the lack of human touch in the chatbot. A participant said that although the chatbot is friendly, there is something about the relation with a real person that is not grasped when chatting with Rosa. When further explaining what she meant by “something,” she used the word *empathy* and said that Rosa cannot see the user in the way a person can. When asked what she would choose between the app and the counselor, she said that she would have chosen the counselor because that would have given her a person to relate to. In particular, she mentioned how difficult she would feel to chat with an app if given a positive genetic test result, meaning, confirmed hereditary cancer risk:

The intimacy of a dialogue or conversation with a human being is not comparable.

Participant 9

Other participants also mentioned how the user is not *seen* by an app. Rosa could not confirm their feelings and ask them how they felt, and they could not ask follow-up questions in return, such as “I don’t quite understand, can you explain in a different way?” Many (7/16) mentioned the lack of eye contact and smiles as something they missed, which prevented them from establishing a relationship with Rosa:

Your doctor can tell if you look worried. This robot won’t be able to do that.

Participant 11

This app was designed with high focus on usability and user-friendliness [4]. However, many (7/16) mentioned different shortcomings related to communicating with this chatbot. The technological shortcomings were not only regarding the performance of the chatbot but also regarding what the participants felt suitable to communicate via such a technological service. Thematically, some issues would not be suitable to learn via a chatbot because of either complexity or sensitivity:

Not everything can be explained by a chatbot.

Participant 9

The unstable ability of the chatbot to understand self-composed questions were mentioned in several interviews. This was mainly solved by altering how they used the chatbot. Instead of asking self-composed questions, they shifted to clicking on read more buttons or suggested readings.

Of the 16 participants, only 1 participant (participant 3) stated clearly that she did not need a tool such as Rosa and that she did not see any value of it compared with Google, apart from the possibility of repeating what had been already said in the counseling session. She further mentioned that she would have valued it if some personal user experiences regarding living with a BRCA mutation had been included. She did not feel more anxious or worried after using Rosa but did not find any comfort in using it either.

#### Trustworthy and True

The fact that the information in Rosa was written by health care personnel gained trust among the participants, and many of them (10/16) conceded that the combination of genetic counseling and using this app comforted them. When one of the participants was asked whether the information was credible, she said that it seemed as if a professional had made it, and she also stressed that she felt the information in Rosa was explained in a simple way, even though some difficult words were used*.* Several participants (8/16) mentioned the feeling of a physician’s language in the chatbot and that reading that type of language made the content trustworthy. They felt that it resembled talking to a nurse or a physician.

Knowing that Rosa was made by health care personnel and provided to them by the health care services was positively mentioned by all the participants (16/16). None of them (0/16) questioned the content of the chatbot or felt the need to double check the information provided by it. Several participants (6/16) stressed that the fact that the information in Rosa coincides with the information one gets from their genetic counselor makes the content more trustworthy, highlighting the value of receiving information via different sources:

When you get the same response in Rosa as you get from your genetic counselor, well it makes you feel extra safe, in a way.

Participant 10

The chatbot’s design was highlighted as something positive. Some of the participants (3/16) spontaneously complemented the avatar as friendly and nicely designed. The interface was described using words such as “harmless,” “professional,” “sweet,” and “like a good person.” Many (12/16) said that there are no stupid questions when chatting with a chatbot, because no one knows what they are asking. The answers provided by Rosa were described as understandable and suitable for all age groups:

It’s written with such closeness that you get it immediately. There are no foreign words. You don’t question what they mean.

Participant 11

Many participants (8/16) expressed that they felt more confident after reading the information they had access to in Rosa. The increased ability to make the right choices for oneself were particularly highlighted. When we asked what a tool such as this app should be able to help a user with, a participant argued that it must help the user feel informed and safe in deciding whether to perform a genetic test and help them understand how that choice may influence their life. Many participants (7/16) mentioned the feeling of being unburdened. They felt that they had a tool that would help them cope with the consequences of learning about potential genetic risk:

It tells you what options you have if you carry the mutation. It supported my decision regarding the surgeries I’m having. It made me even more sure that I’m going to do it.

Participant 14

Many (11/16) stated the value of having access to this app early in the process, especially before genetic testing, but Rosa was also highlighted as a valuable tool for information later in the process. They valued that Rosa could be used to answer questions by family members, ensuring that what they communicated was the truth:

Everything is there; How to tell your children, how to move forward, what is important to remember, how to tell people around you. And that is the difficult part, how to tell others. But then you have your answers her. Instead of using your own words.

Participant 16

All participants (16/16) were asked how they reacted toward reading about a potentially sensitive topic such as hereditary cancer via a chatbot. None of the participants (0/16) said that they felt more stressed, anxious, or nervous when using Rosa, which was a major concern before this study. In contrast, several (12/16) said that the facts are not scary.

## Discussion

### Principal Findings

In this study, we wanted to explore 2 aspects of chatbot use in healthy patients at risk of hereditary cancer—first, their perceived usability of the chatbot and their trust in the technology and, second, how they perceived to obtain potentially sensitive genetic information from a chatbot. What became very evident early on was the positive attitude and willingness in users to engage in Rosa. The concept, “Anytime, anywhere,” focuses on the availability and accuracy of the information that make the chatbot very relevant. Although none of the participants (0/16) expressed having negative feelings when chatting with Rosa, such as increased worry, none of them (0/16) wanted Rosa as their only access to genetic information. The counseling aspects achievable only through human interaction were highlighted as essential, with the chatbot being a valuable addition, as described in the concept, “In addition, not instead.” Using Rosa along with genetic counseling helped them feel safe in the choices ahead, as they could repeat information and ask questions they had forgot during the counseling session. This setting was mentioned as stress relieving and supportive and formed the concept, “Trustworthy and true.”

Overall, 2 aspects pervade all interviews—what this digitalization removes and what it adds. Communicating with an app takes away the feeling of interaction or the humanity of the conversation. The participants do not feel seen or acknowledged. Rosa cannot ask personal follow-up questions that would have been natural in a human-to-human conversation. The “You look worried?” or “I get the feeling I am not expressing myself properly?” and similar questions that genetic counselors may ask if the patient look worried or confused will not be asked. The intimacy of a human conversation is missing, and the conversation with Rosa is described in neutral, sober terms. Although they get medically correct information provided by health care personnel, in a format available anytime and anywhere, it is not comparable with human interaction.

In contrast, what they gain is described in words often used when expressing increased empowerment. Expressing a feeling of being more confident in the process about to be initiated; the feeling of stress relief in the situation; a lowered level of worry because of access to quality-assured information; and the recognition that they, after all, will cope, all point in a direction of increased empowerment. Although empowerment was not specifically assessed during the interviews, the words the participants used to describe how interaction with Rosa affects them resemble words describing empowerment. They share that they gain knowledge about genetic testing and its consequences and that Rosa helps them to make the right decision and to feel confident in that choice. In addition, Rosa is available 24/7, allowing them to access information and support whenever they need it. They have a tool for upcoming queries for either themselves or their family, and the data showed that they have no feelings of stress or worry related to using Rosa.

It has previously been argued that digital health tools have an empowering potential, as they provide individuals with the information they need to be proactive about their own health [24]. This focus on the empowering potential of digital health tools has also been criticized [24,25]. Digital health tools may place too much responsibility on the patient’s self-surveillance and promote conformity rather than autonomy, leading Morley and Floridi [25] to argue toward shifting from focusing on empowerment to focusing on the value of digital companionship. All our participants (16/16) highlighted the value of having a digital companion in the genetic testing process, in particular, related to gathering information about the potential life-changing choices when having a BRCA mutation trigger. Rosa was a safe place to engage in the sensitive aspects of genetic testing. The feeling of not being alone is a valued aspect of digital companionship [26]. A participant described Rosa as having a friend beside them, one who is there for the whole ride. She further said that she felt as if she was ahead of the situation when having access to Rosa, thus describing a feeling of being prepared. The support felt by having a place to seek tailored information specific to one’s own need highlights the power that lies in companionship, real or digital. The real benefit of digital tools is realized when users and health care personnel are given the ability to use them to navigate in a shifting health situation, supporting both parties in the exchange of information. Morley and Floridi [24] stress that naming it as companionship rather than empowerment avoids placing too much responsibility of own health on the individual, for which empowerment is criticized. In addition, assuming that information equals empowerment is not necessarily valid [24]. Providing the right information at the right time, as Rosa does, is congruent with the general perception of important traits in digital companions [26].

When building Rosa, one of our main concerns was whether having information regarding delicate topics such as risk of cancer via a chatbot would be perceived as cold or insensitive. None of the participants (0/16) confirmed this, consistent with similar studies on chatbot use in health care [27,28]. Rosa was viewed as efficient and supportive. An overall initial aim when building Rosa was to provide correct and relevant genetic information to the patients, accessible at their own time and pace [4]. The concept, “Anytime, anywhere,” confirms this aspect. Our participants valued the possibility the chatbot provided for preparing for upcoming appointments at the hospital and repeating information afterward. This stresses the value of having access to a chatbot throughout the genetic counseling and testing trajectory.

Both health care personnel and the patients must be able to rely on the information provided by chatbots delivered by the health care services. There is no tolerance of faulty information, as conversational agents such as OpenAI ChatGPT [29] may provide. We have solved this by using a platform of precomposed answers written by health care personnel. This approach calls for high commitment both in making the database and maintaining it. We found that our participants prefer and trust digital services made by health care personnel and value health care personnel involvement specifically. This tells us that we need to be hands on in the development, testing, evaluation, and implementation process of new technologies replacing or supplementing our services. Rosa was in use for several months after the last patients had been included, indicating that the chatbot has been useful also after the genetic test result was revealed and the patient’s interaction with the ordinary genetic services had ended. This reinforces the importance of such tools in genetic services particularly, and in health care generally, being developed and maintained in strong companionship with qualified health care personnel.

The presence of a professional human and what that entails, for example, eye contact and being asked appropriate follow-up questions, is incomparable with a chatbot conversation. The technology is developing fast, and the companionship and social support many feel when interacting with commercial conversational agents and “virtual friends” [30] may also be transferrable to health chatbots in the future. This adds another aspect to chatbot use in health care, and another level of benefits for the patients may be revealed. On the critical side, the availability of well-performing and highly credible chatbots in the society in general may lead patients to trust the chatbot’s content without criticism of the source. Our participants confirmed this. None (0/16) felt the need to double check the information given by Rosa. This leaves a responsibility for health care personnel and the health care services to provide quality-assured bots easily recognizable as coming from the health care services, thus safeguarding our patients and preventing misinformation and faulty advice.

The high fallback rate in open-ended questions is a challenge for further investigation. Previous studies have pointed to the limited use of open-ended questions and users preferring the presented predefined options [31]. This highlights the importance of carefully selecting relevant follow-up questions and reading more suggestions to keep the user engaged in the app [31]. This feature was also mentioned as crucial by all participants (16/16) in our study, not particularly because of the performance of the chatbot but because of the complexity of the topic, as many found it difficult to compose the right question to extract the right answer from the database.

Chatbots operating with a closed database will need constant update and maintenance, requiring continuous human resources. Any questions that trigger the fallback mechanism must be handled manually, either by assigning them the appropriate preexisting answer within the chatbot or by providing a new and accurate answer that can be used for similar questions in the future. To ensure the continued maintenance of Rosa, we have engaged genetic personnel from all health regions in Norway, who take turns in performing this work as part of a dedicated team. Without this continuous work, the chatbot will quickly be outdated, and the dynamic potential in the tool as an easy-to-use, reliable source of quality-assured, updated information is lost. A well-performing chatbot is important to keep the tool relevant both for new users and existing users whose needs will change over time. The ability to provide accurate and relevant information in a way that meets the users’ expectations of a good answer is crucial for a chatbot’s trustworthiness [32].

Our findings are very consistent with a recent study aiming at identifying the “sweet spot” for chatbots in medical genetics [33]. The informants in their study [33] were not evaluating a specific chatbot but rather asked to share their opinions regarding implementing chatbots in genetic services. They argue that the moderately complex issues are where the chatbot may close a gap. For simple tasks, other low-threshold services will be more efficient (booking portals, etc), whereas for complex tasks, such as specific, nuanced, and personal questions, a chatbot has the risk of misunderstanding or being wrong. The moderately complex tasks, where they argue that “sweet spot” lies, are exemplified as general genetic information, disease information, and providing status updates and information about next steps [33]. These were tasks characterized by a high return on investment, meaning that the input of time and energy was worth the output received. Rosa fits into this “sweet spot” and may explain the overall positive feedback we had. The coinciding finding between the study by Luca et al [33] and our study regarding chatbots serving best as a supplement and not as a replacement to genetic counseling is worth highlighting. Patients both in cancer genetics and clinical genetics in general have the same attitudes toward chatbots as a welcomed supplement, as long as it hits the “sweet spot” and does not act alone. Our participants emphasized in addition the importance of being able to repeat the information that had been provided during genetic counseling through the chatbot and the possibility to use the chatbot to prepare for upcoming genetic counseling, thus tailoring the conversation. This ability to repeat and prepare may add to this “sweet spot” and underpin the value of digital companionship.

### Strengths and Limitations

Results from qualitative studies are not intended to be generalizable. Our data are obtained from healthy, at-risk individuals in families with breast and ovarian cancer. We had a broad variety of participants in our final sample selected for in-depth interviews. We recruited patients from all over the country to participate; we ensured that we have both men and women with and those without a BRCA mutation and have all age groups represented to reflect the width of our total sample. With a qualitative approach, we ensure a thorough evaluation of the chatbot and may elaborate about topics according to the participants wishes. Using the SDI model for analyses of data where no codes are generated in advance allow findings to emerge from the dominant themes inherent in the raw data. The deductive loops provide quality assurance before moving to the next step, allowing the data set to speak, thus keeping the codes empirical until the final step where concepts are generated. This reduces the possibility of the authors influencing the final concepts with their own preconceptions.

As 3 authors of this paper are either practicing genetic counselors or medical geneticists, the SDI method was chosen to minimize the influence of authors’ expectations on the findings. Furthermore, the research group was deliberately expanded by adding 2 experts in qualitative methodology and ensuring the quality of the analyses. All study meetings had a focus on preconceptions, and every step in the SDI approach was validated by all to enhance reflexivity. For this specific study, the researchers’ in-depth knowledge about genetic counseling proved to be an advantage during the interviews. The participants could ask clarifying questions regarding the chatbot’s content if they had any and share potential emotions regarding the genetic process they had been through knowing that health care professionals interviewed them. This may also have been a bias in the study, as knowing the researchers’ affiliations in the genetic field may have influenced how the participants spoke about the chatbot. It is possible they were reluctant to be honest or felt obliged to speak more positively regarding the product than they would have if the researchers were outside the genetic field. The research group was aware of this during the analyzing process and considered the most positive comments with caution and ensured to highlight the negative feedback for balance.

Every study with clinical samples has the risk of sampling bias, and a central limitation of our study is that we have no information about those who did not return the consent form. The oldest participant was aged 55 years, we had no one of non-Norwegian culture, and all participants had education at vocational level or higher. We may have selected a subsample of highly literate patients who enjoy technological developments, hence missing the critical voices. In contrast, the information provided by the chatbot is composed in lay language with high level of user involvement, ensuring that the information is accessible to almost all users [4]. Therefore, worry or confusion owing to contradictory advice or misunderstandings are unlikely. We had a participant who found Rosa to be redundant and labeled herself as critical of it; however, she had no trouble in maneuvering the app and did not feel anxious or worried when using it. Another limitation is the registration of chatbot use. Owing to privacy regulations, we were unable to link the participants with the entries in the app, resulting in a lack of precise data about the number of entries or log-in time per participant. Therefore, all participants were asked during the interviews about their use of the chatbot.

As we were unable to conduct face-to-face interviews owing to the COVID-19 pandemic, the interviews were conducted via video. We may have missed follow-up questions or comments that would otherwise have been provided if the interview had been conducted face to face, with the intimacy that follows such a setting. However, the participants are quite unified in their feedback, and their experiences using Rosa are comparable with those mentioned in previous studies. With more studies confirming the place and role for chatbots in health care in general, and genetic services in particular, the validity of the findings increases.

### Implications for Practice and Future Studies

Chatbot use in clinical practice has the potential to contribute to uniform information for everyone, regardless of residence and access to specialized health care personnel, without adding discomfort or worry. It is likely to assume that chatbots are here to stay. The popularity of chatbots such as the OpenAI ChatGPT [29] and web-based friends suggests that there is a future for chatbots. Health care chatbots may contribute to hybrid health services and equal access to health care by being a digital companion that patients appreciate. We know that patients want and expect hybrid health services, combining digital and in-person services [3,16]. Hybrid health care models make patients feel engaged and empowered [16]. A user-friendly health service using the best from both the digital and in-person services is a future goal to strive for, which can only be achieved after comprehensive studies involving both patients’ perspective and health care services’ perspective.

There is a need to elaborate on drivers of and barriers to chatbot interaction in a genetic setting, as has also been mentioned in previous studies [11]. However, drivers of and barriers to chatbot *implementation* are equally important. To achieve a seamless transition between the digital and in-person services, we postulate that future studies of chatbots in health care must focus on the 3 Is—interaction, integration, and implementation—from both the patients’ perspective and the health care services’ perspective.

### Conclusions

Our results indicate that a genetic information chatbot contributes to uniform information for our patients, regardless of geographical location. The 24/7 availability of quality-assured information, tailored to the specific situation the patient is facing, has had a reassuring effect on the participants in our sample. The chatbot is a tool available anytime and anywhere, not replacing genetic counseling but serving as an addition, providing reliable content the patients can trust. It was consistent across concepts that Rosa was a tool for preparation and repetition. We argue that this adds to the “sweet spot” that genetic chatbots need to fulfill. All participants (16/16) denied increased worry after reading about hereditary breast and ovarian cancer and genetic testing in Rosa, implying chatbots to be a well-suited digital companion to genetic counseling with an empowering potential.

## Appendix

Multimedia Appendix 1 English translation of the semistructured interview guide.

## Abbreviations

AI: artificial intelligence
SDI: stepwise-deductive inductive

## Data Availability

The data sets generated and analyzed during this study are not publicly available owing to privacy regulations but are available from the corresponding author upon reasonable request.
