# Supplementary material for: Evaluation of the Rosa Chatbot Providing Genetic Information to Patients at Risk of Hereditary Breast and Ovarian Cancer: Qualitative Interview Study
Source: J Med Internet Res. 2023 Sep 1;25:e46571. doi: 10.2196/46571 (PMC10504626; doi:10.2196/46571)
Supplement: Multimedia Appendix 1 [file jmir_v25i1e46571_app1.docx]

**Appendix 1: In-dept video interviews with healthy individuals with a family history of breast and ovarian cancer who have tried out the chatbot ROSA**

**Introduction**:

• Introduce ourselves and the project. Clarification of expectations.

• Topic of conversation: usability, usefulness, trust in the tool, and thoughts on the impact of using this type of technology

• Recording device, anonymization, use of the information shared, confidentiality agreement

• "Ground rules" for the conversation - there are no right or wrong answers, you are the expert here

**Interview guide**

The interview guide serves as a starting point, and participants will be given the opportunity to emphasize what they find important in this context.

**Part 1: Usability, perceived usefulness, and trust in information**

**I- Relation to technology and the situation you are in:**

To what extent would you say the situation you are in (the genetic process) concerns you?

Did you have any expectations of what an app like Rosa could do/contribute?

What did you know about chatbots before?

**II- Usability; How did you experience navigating through the bot?**

Probes:

How did you like the menu in the upper right corner?

How did you like the chat function?

Probes:

Did you prefer typing in questions or choosing from the suggested buttons provided by Rosa?

How was it to find answers to what you were wondering about?

Did you experience receiving answers that didn't match the question you asked?

How did you feel about receiving the response "I don't understand," and how did you proceed from there?

Is there anything you would like to highlight as positive about using Rosa?

Was there anything particularly technically challenging or something that could have worked better?

**III- Usefulness; Did you find it useful to use Rosa?**

Probes:

Were there any benefits to having access to Rosa?

What did you find useful and not useful about the app itself?

What do you think is important for a technological tool like Rosa to be able to do or assist with?

Can Rosa help you in decision-making processes (such as breast removal or genetic testing), and if so, how?

**IV-Trust and care - How did you experience the responses from Rosa, and is she trustworthy and caring?**

Would you say that you had a conversation with Rosa?

Probes:

Did you reflect on the fact that you were communicating with a machine?

What did you like/dislike about the way Rosa formulated her responses?

How did you experience the "personality" of Rosa? Machine-like vs. "human touch."

To what extent was she understandable?

To what extent was she believable?

**V- Empowering the user; Has the use of Rosa had any influence on your decisions?**

Probes:

Do you feel that Rosa has affected your ability to take action/make choices? How?

Do you feel that Rosa has influenced your insight/understanding of the situation you are in?

Has the information you received from Rosa created new needs/questions? What would you like to have more/less of?

**Part 2: How does Rosa impact the user, and for whom should tools like Rosa be beneficial?**

**VI- Experiences with Rosa**

Was there anything about Rosa that particularly made you feel taken care of? Anything specifically important?

How did Rosa influence you?

Probes:

Was there any information that you found difficult or uncomfortable in any way?

Did it affect you that you communicated about this topic through an app rather than face-to-face? What is different?

Do you think that Rosa influenced your relationship with the healthcare system in any way? (e.g., more/less responsibility for your situation, proximity/distance, more/less alone with decisions and concerns, etc.)

Would you say that Rosa in any way contributed to changing the situation you were in - if so, how?

**VII- Thoughts on future use of digital tools in healthcare**

Do you think that Rosa and similar digital tools are beneficial for us and something we should offer in the future of healthcare?

Probes:

Which values do you think we, as a society, uphold by allowing information/communication to occur through digital technology? What/whom do we do it for?

What would you have thought if your appointment with a genetic counselor had been replaced by a chatbot or online service? What assistance might you have missed out on?

Do you think that Rosa or similar tools could replace face-to-face consultations in the future? Is there a limit to what can be treated/consulted through online services?

**Summary:**

Is there anything you would like to add beyond what we have discussed?

Summarize the main themes of the discussion, allow for comments if desired.
